# Association of tyrosine hydroxylase *01* (*TH01*) microsatellite and insulin gene (*INS)* variable number of tandem repeat* (*VNTR) with type 2 diabetes and fasting insulin secretion in Mexican population

**DOI:** 10.1007/s40618-023-02175-4

**Published:** 2023-08-25

**Authors:** J. Berumen, L. Orozco, H. Gallardo-Rincón, E. Juárez-Torres, E. Barrera, M. Cruz-López, R. E. Benuto, E. Ramos-Martinez, M. Marin-Madina, A. Alvarado-Silva, A. Valladares-Salgado, J. J. Peralta-Romero, H. García-Ortiz, L. A. Martinez-Juarez, A. Montoya, D. A. Alvarez-Hernández, J. Alegre-Diaz, P. Kuri-Morales, R. Tapia-Conyer

**Affiliations:** 1 Facultad de Medicina, Unidad de Investigación en Medicina Experimental, Universidad Nacional Autónoma de México, 06720 Mexico City, México; 2 Laboratorio de Inmunogenómica y Enfermedades Metabólicas, Instituto Nacional de Medicina Genómica, Secretaria de Salud, 14610 Mexico City, México; 3 Departamento de Soluciones Operativas, Fundación Carlos Slim, 11529 Mexico City, Mexico; 4 Centro Universitario de Ciencias de la Salud, Universidad de Guadalajara, Sierra Mojada 950, 44340 Guadalajara, Jalisco México; 5 Laboratorio Huella Génica, Unidad de Diabetes, 06600 Mexico City, Mexico; 6 Unidad de Investigación Médica en Bioquímica, Hospital de Especialidades, Centro Médico Nacional Siglo XXI, Instituto Mexicano del Seguro Social, 06720 Mexico City, México; 7 Center for Humanitarian Health, Johns Hopkins Bloomberg School of Public Health, Baltimore, MD USA; 8 Facultad de Medicina, Universidad Nacional Autónoma de México, Coyoacán, 04510 Mexico City, México; 9 Proyecto OriGen, Instituto Tecnologico y de Estudios Superiores de Monterrey, Monterrey, México

**Keywords:** Diabetes, *INS*, Insulin, rs689, *TH01*

## Abstract

**Purpose:**

A variable number of tandem repeats (VNTR) in the insulin gene (*INS*) control region may be involved in type 2 diabetes (T2D). The *TH01* microsatellite is near *INS* and may regulate it. We investigated whether the *TH01* microsatellite and *INS* VNTR, assessed via the surrogate marker single nucleotide polymorphism rs689, are associated with T2D and serum insulin levels in a Mexican population.

**Methods:**

We analyzed a main case–control study (n = 1986) that used univariate and multivariate logistic regression models to calculate the risk conferred by *TH01* and rs689 loci for T2D development; rs689 results were replicated in other case–control (n = 1188) and cross-sectional (n = 1914) studies.

**Results:**

*TH01* alleles 6, 8, 9, and 9.3 and allele A of rs689 were independently associated with T2D, with differences between sex and age at diagnosis. *TH01* alleles with ≥ 8 repeats conferred an increased risk for T2D in males compared with ≤ 7 repeats (odds ratio, ≥ 1.46; 95% confidence interval, 1.1–1.95). In females, larger alleles conferred a 1.5-fold higher risk for T2D when diagnosed ≥ 46 years but conferred protection when diagnosed ≤ 45 years. Similarly, rs689 allele A was associated with T2D in these groups. In males, larger *TH01* alleles and the rs689 A allele were associated with a significant decrease in median fasting plasma insulin concentration with age in T2D cases; the reverse occurred in controls.

**Conclusion:**

Larger *TH01* alleles and rs689 A allele may potentiate insulin synthesis in males without T2D, a process disabled in those with T2D.

**Supplementary Information:**

The online version contains supplementary material available at 10.1007/s40618-023-02175-4.

## Introduction

**Fig. 1:**
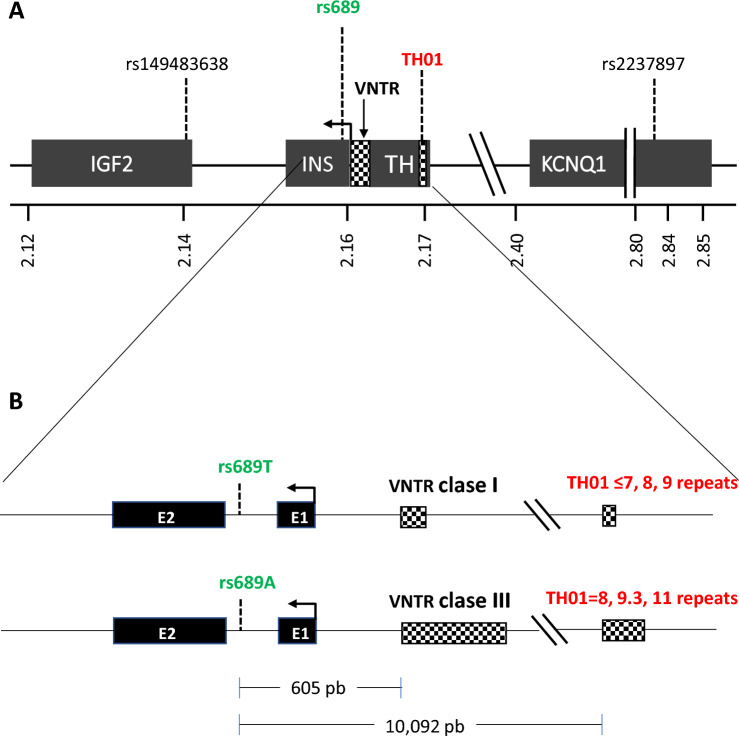
Genomic map of region 11p15.5. Panel **A** shows the locations of genes *IGF2*, *INS*, *TH*, and *KCNQ1* and the markers SNP rs689, VNTR *INS*, and *TH01* microsatellite. The positions are based on the human genome version GRCh38. The square arrow indicates the direction and site of initiation of gene transcription. Panel **B** shows a zoom of the *INS*-*TH* region indicating the distance between the rs689 and VNTR and *TH01* markers and the alleles of rs689, *INS* VNTR and *TH01* linked in European and Japanese populations (rs689/*INS* VNTR) and found in this report (rs689/TH01). *bp* base pairs, *SNP* single nucleotide polymorphism, *VNTR* variable number of tandem repeats

In the control region of the *INS* gene, there is a variation in the number of tandem repeats (VNTR) polymorphism at 390 base pairs (bp) from the start of *INS* transcription (Fig. 1A) that is associated with type 1 diabetes [1–3]. The *INS* VNTR consists of a sequence of 14–15 nucleotides (5′-ACAGGGGTGTGGGG-3′) repeated in tandem [4] and has been shown to participate in the regulation of *INS* in healthy individuals [5–7]. *INS* VNTR alleles are categorized according to the number of repeats in class I (26–63), class II (64–140), and class III (141–209). While class I *INS* VNTR alleles are considered to confer a risk of developing type 1 diabetes, class III alleles are generally associated with protection [4]. Unlike class III alleles, class I alleles have been associated with increased insulin production [8, 9].

Although *INS* VNTR has been associated with type 2 diabetes (T2D) [10–13], the results remain controversial. Moreover, because most studies were conducted in European populations, there is little evidence in Latin American populations. There could be different associations between *INS* VNTR and T2D in the latter population, as polymorphisms in *SLC16A11*, *INS-IGF2*, and *HNF1A* genes have previously been reported to have strong associations with T2D in Mexicans but not in European populations [14, 15].

The *INS* VNTR is complex; therefore, a surrogate marker, the single nucleotide polymorphism (SNP) rs689, has been frequently used [13]. This marker is found within intron 1 of *INS* and is in complete linkage disequilibrium with the *INS* VNTR in European populations (allele A is associated with class III alleles; allele T, with class I alleles; Fig. 1B). In addition, the tyrosine hydroxylase (*TH)01* microsatellite, a tetranucleotide (AATG) repeated 3–11 times in tandem, is also located relatively close to *INS* (approximately 9800 bp; Fig. 1A). It is located within the *TH* gene that is in partial linkage imbalance with *INS* VNTR in European [1, 16] and Japanese [17] populations. In addition, in vitro experiments have demonstrated that *TH01* has enhancer functions, i.e., it can regulate gene expression at a distance, a common attribute for microsatellites [18], and could potentially influence *INS* expression [19].

Interestingly, although *TH01* is associated with obesity [20], hypertension [21, 22], coronary heart disease [23], metabolic syndrome, and high triglyceride levels [24, 25], its association with T2D has not been explored. Therefore, this study was conducted to investigate whether the *TH01* microsatellite and *INS* VNTR (through SNP rs689) are associated with T2D and fasting plasma insulin concentration in the Mexican population. Since in a previous study in Mexican population [26] we found important differences in the association of genes with T2D between males and females and between individuals diagnosed early (≤ 45 years) and late (≥ 46 years), in this paper we also explore whether *TH01* and rs689 have a different influence on the development of T2D between sexes and age of presentation. The main study was a case–control study that used logistic regression models to calculate the risk (odds ratio; OR) conferred of each locus for T2D in a Mexican population; a clinical replica case–control study and a cross-sectional study were also conducted.

## Materials and methods

### Sample selection and study design

Both *TH01* and rs689 were analyzed in the main case–control study, which included individuals of the Diabetes in Mexico Study (DMS), the details of which have been previously described [14, 15]. Briefly, participants were recruited between November 2009 and August 2013 from two tertiary-level hospitals in Mexico City; T2D was diagnosed based on the American Diabetes Association (ADA) [27] criteria. A total of 988 cases (unrelated individuals aged > 20 years, with a previous diagnosis of T2D or fasting blood glucose > 125 mg/dL) and 998 controls (healthy individuals aged > 50 years with fasting blood glucose < 100 mg/dL); all participants were self-recognized as Mexican mestizo.

For the replication of rs689, a case–control study included 593 cases of T2D and 595 controls (recruited between January 2014 and January 2015 from a tertiary care hospital in Mexico City). Cases consisted of individuals previously diagnosed with T2D according to the ADA criteria, who agreed to participate and were continuously recruited during routine medical visits. Controls were individuals aged ≥ 50 years, who attended the same clinics for reasons other than T2D, had fasting blood glucose < 100 mg/dL, and agreed to participate in the study.

Another replica of rs689, a population-based cross-sectional study, was conducted on 1172 from 1914 patients recruited between July and December 2017 from a hospital in Puebla, Mexico. Healthy individuals were invited to participate through flyers distributed in the hospital’s neighborhood. The procedures used for *TH01* and rs689 SNP genotyping are in Online Resource 1.

### Ethics

The protocol for the main study was approved by the local ethics committee of each study site and the Federal Commission for the Protection Against Health Risks (COFEPRIS) (CAS/OR/CMN/113300410D0027-0577/2012). The clinical replica study was approved by the Ethics and Research Committees of the Comisión Nacional de Investigación Científica of the Instituto Mexicano del Seguro Social (IMSS R-2014-785-005), while the cross-sectional study was approved by the Ethics and Research Committees of the Hospital General de Puebla “Ignacio Romero Vargas” (68/ENS/INV/REV/2017). All protocols complied with the Declaration of Helsinki and local ethical guidelines for clinical studies in Mexico. Written informed consent was obtained from all participants.

### Statistical analyses

For the sample size calculation, multivariate logistic regression (MLR) models were considered to have good performance when a baseline of 100 cases (i.e., the univariate logistic regression models) and 15 additional cases for each variable were introduced into the model [28]. Given that only two variables were to be introduced in the MLR models, we calculated that a minimum number of 115 cases were needed in the comparison groups of the main case–control study in which both loci were studied.

Logistic regression models were used to calculate the risk or protection conferred by the *TH01* and rs689 loci for T2D development in the whole sample and stratified by sex and the median (45 years) age of T2D presentation (≤ 45 and ≥ 46 years). Cases diagnosed with T2D at ≤ 45 years were compared with controls aged ≤ 54 years, and cases diagnosed with T2D at ≥ 46 years were compared with controls aged ≥ 55 years. The statistical significance of differences in the distribution of genotypes between the observed and expected results was calculated using the chi-square test according to the Hardy–Weinberg law, and the linkage disequilibrium between *TH01* and rs689 SNP alleles, using Arlequin (version 3.5.2.2; http://cmpg.unibe.ch/software/arlequin35/) [29].

A post-hoc power analysis was performed for each model using the G^*^Power software version 3.1.9.7 (https://www.psychologie.hhu.de/arbeitsgruppen/allgemeine-psychologie-und-arbeitspsychologie/gpower), considering the sample size, odds ratio (OR), probability of the event in the control group, and an α = 0.05 [30]. All statistical tests were two-sided, and the significance level was set at *P* < 0.05. The statistical analyses were conducted using SPSS software version 25 (IBM Corp., Armonk, NY, USA). Additional information on the statistical methods is provided in Online Resource 1.

## Results

### Participants and demographic characteristics

The 53.3%, 60.6%, and 73.5% of participants were female in the main case–control (n = 1986), replica case–control (n = 1188), and cross-sectional (n = 1172) studies, respectively. In both case–control studies, nearly 50% of participants were T2D cases, whereas in the cross-sectional study, the prevalence of T2D was 5.4%. At enrollment, the mean (SD) age of controls vs. cases and the mean age at the time of T2D diagnosis were similar across the studies (Table 1).

**Table 1:** Participant demographic characteristics (*n* = 4346)

Variable	Female		Male		Both sexes	
	Control	Cases	Control	Cases	Control	Cases
Main case–control study (*n* = 1986): mean ± SD (*n*)						
Age (years)	59.7 ± 11.2 (535)	55.4 ± 12.1 (524)^¶^	58.6 ± 11.4 (463)	55.8 ± 11.4 (464)^§^	59.2 ± 11.3 (998)	55.6 ± 11.7 (988)^¶^
BMI (kg/m^2^)	28 ± 4.9 (531)	29.6 ± 5.5 (524)^¶^	26.9 ± 4.1 (457)	28.7 ± 5 (459)^¶^	27.5 ± 4.6 (988)	29.2 ± 5.3 (983)^¶^
Waist (cm)	93.3 ± 11.3 (348)	97.7 ± 11.6 (409)^¶^	93.4 ± 10.5 (310)	98.7 ± 12.7 (279)^¶^	93.4 ± 10.9 (658)	98.1 ± 12 (688)^¶^
Hip (cm)	103.6 ± 11 (327)	106.5 ± 11.5 (408)^§^	98.9 ± 7.9 (298)	101.7 ± 10.8 (275)^§^	101.4 ± 9.9 (625)	104.6 ± 11.5 (683)^¶^
WHR	0.9 ± 0.07 (327)	0.92 ± 0.06 (407)^§^	0.94 ± 0.06 (298)	0.97 ± 0.06 (275)^¶^	0.92 ± 0.07 (625)	0.94 ± 0.07 (682)^¶^
Age at T2D diagnosis (year)		45.9 ± 10.6 (524)		46.2 ± 10.9 (464)		46 ± 10.8 (988)
Years with the disease		9.4 ± 8.4 (524)		9.6 ± 9 (464)		9.5 ± 8.7 (988)
Replica case–control study (*n* = 1188): mean ± SD (*n*)						
Age (years)	53.4 ± 9.6 (423)	57 ± 8.8 (297)^¶^	55.8 ± 11 (172)	57.3 ± 10.2 (296)	54.1 ± 10.1 (595)	57.1 ± 9.5 (593)^¶^
BMI (kg/m^2^)	28.9 + 5.1 (423)	30.6 + 5.1 (297)^¶^	28.9 + 4.3 (172)	28.3 + 4.3 (296)	28.9 + 4.9 (595)	29.5 + 4.9 (593)^†^
Waist (cm)	92 + 11.2 (423)	96.4 + 10.9 (297)^¶^	98.3 + 10.3 (172)	98.5 + 11 (296)	93.8 + 11.3 (595)	97.4 + 11 (593)^¶^
Hip (cm)	105.2 + 11.9 (423)	107 + 11.5 (297)^†^	102.7 + 8 (172)	101.1 + 8.2 (296)*	104.5 + 11 (595)	104.1 + 10.4 (593)
WHR	0.89 + 0.4 (423)	0.9 + 0.1 (297)	0.96 + 0.1 (172)	0.97 + 0.1 (296)^‡^	0.91 + 0.3 (595)	0.94 + 0.1 (593)*
Age at T2D diagnosis (year)		45.3 ± 7 (297)		44.3 ± 7.5 (296)		44.8 ± 7.3 (593)
Years with the disease		11.7 ± 7.8 (297)		12.9 ± 7.5 (296)		12.3 ± 7.7 (593)
Cross-sectional study (*n* = 1172^a^): mean ± SD (*n*)						
Age (years)	49.3 ± 10.7 (789)	49 ± 11.7 (72)	48.8 ± 11.1 (279)	49.5 ± 10.9 (32)	49.1 ± 10.8 (1068^d^)	49.2 ± 11.4 (104)
BMI (kg/m^2^)	28.5 ± 4.7 (789)	31.1 ± 4.6 (72)^§^	28.6 ± 4 (279)	30.7 ± 7.3 (32)^§^	28.5 ± 4.5 (1068)	31 ± 5.5 (104)^¶^
Waist (cm)	91.2 ± 15.4 (789)	98.1 ± 15.4 (72)^§^	95.3 ± 14.8 (279)	101.8 ± 12.2 (32)^§^	92.3 ± 15.3 (1068)	99.2 ± 14.6 (104)^¶^
Age at T2D diagnosis (year)^b^		49.8 ± 11.7 (63)		48.8 ± 10.6 (29)		49.5 ± 11.3 (92)
Years with the disease^c^		≈0		≈0.7		≈0

*SD* standard deviation

*p < 0.1; †p < 0.05, ‡p < 0.01, §p < 0.001, ¶p < 0.000

^a^1914 individuals were recruited but only 1172 were explored for rs689 locus

^b^92 newly diagnosed cases were included and age at recruitment was used. The age at diabetes diagnosis of the 12 individuals with type 2 diabetes diagnosed before recruitment was not collected

^c^Difference between mean age of all 104 cases less than the mean age of 92 newly diagnosed cases

^d^This control group includes individuals with pre-diabetes (*n* = 529; A1c ≥ 5.7 to < 6.5), and non-diabetic individuals aged ≥ 40 years (*n* = 539; A1c < 5.7)

### Frequency and association of alleles and genotypes of *TH01* with T2D using regression models

**Fig. 2:**
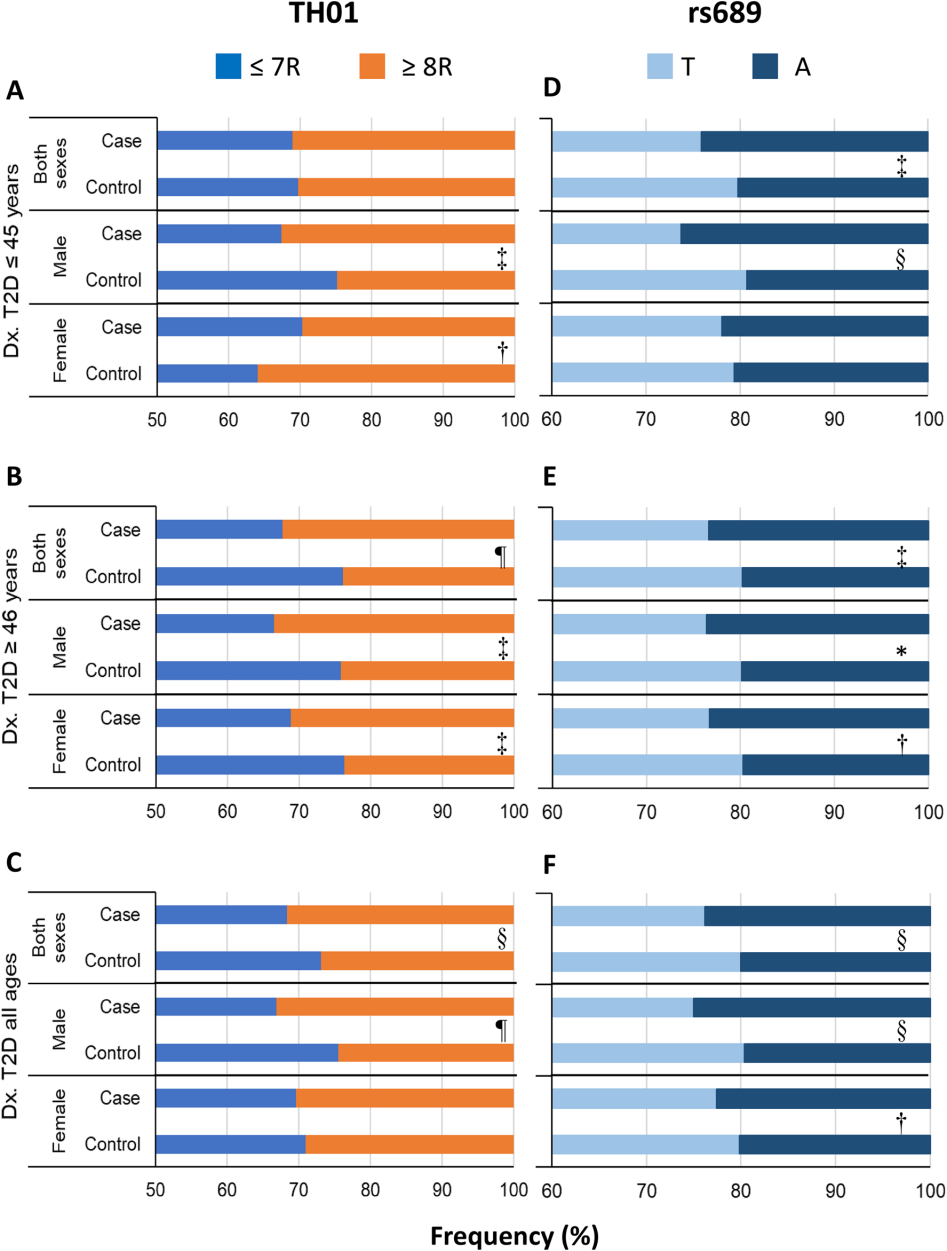
Comparison of allelic frequencies of *TH01* and rs689 markers between cases and controls by sex and age of T2D presentation. For *TH01*, only the main case–control study was explored (*n* = 1986; 3972 chromosomes), whereas, for rs689, the main and replica case–control and cross-sectional studies were explored as a pool (*n* = 4341; 8682 chromosomes). Cases diagnosed with T2D at ≤ 45 years were compared with controls aged ≤ 54 years, and cases diagnosed with T2D at ≥ 46 years were compared with controls aged ≥ 55 years. ≤ 7R = alleles with 3, 4, 5, 6, and 7 repeats, and ≥ 8R = alleles with 8, 9, 9.3, and 11 repeats. *Dx* diagnosis, *OR* odds ratio, *SNP* single nucleotide polymorphism, *T2D* type 2 diabetes. The chi-square test was used to assess the statistical significance; statistically significant values are labelled as *p < 0.1; ^†^*p* < 0.05, ^‡^*p* < 0.01, ^§^*p* < 0.001, ^¶^*p* < 0.0001

**Fig. 3:**
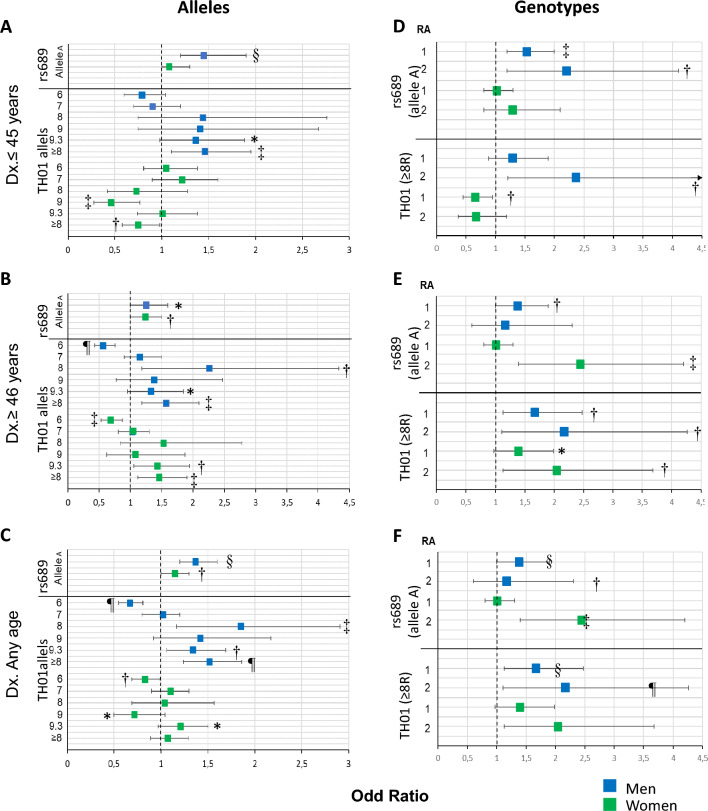
Association study with alleles and genotypes of *TH01* and rs689 markers stratified by sex and age of T2D presentation. See the legend of Fig. 2. Univariate logistic regressions were performed in the whole sample and stratified by sex and age of T2D presentation. The Wald test was used to assess the statistical significance using the Enter method; statistically significant values are labelled with as follows: *p < 0.1; ^†^*p* < 0.05, ^‡^*p* < 0.01, ^§^*p* < 0.001, ^¶^*p* < 0.0001. *CI* confidence interval, *OR* odds ratio. The power (1 − β error probability) > 0.99 for both markers, with *p* < 0.05 in all groups

The frequency of alleles and genotypes of *TH01* was only assessed in the main study and summarized in Fig. 2A–C and Online Resource 2. Though we identified 9 out of 11 alleles reported for the *TH01* microsatellite [31], only five (6, 7, 8, 9, and 9.3) had a frequency ≥ 5% in cases or controls. Allele 6 had a significant protective effect towards T2D diagnosed at ≥ 46 years, with a 32.0% reduced risk in females and 44.0% in males (Fig. 3B); in addition, allele 9 had a protective effect for T2D in females diagnosed at ≤ 45 years (54.0%) (Fig. 3A). The opposite effect was observed for allele 8, which conferred a 2.26-times greater risk for T2D diagnosed at ≥ 46 years in males, and allele 9.3, which conferred a 1.43-times increased risk in females diagnosed at ≥ 46 years (Fig. 3B) and a 1.34-times increased risk in males diagnosed at either age cutoff (Figs. 3C). The differences between alleles were seen more clearly when pooled by size; large alleles (L) with ≥ 8 repeats (R; 8, 9, 9.3, and 11) conferred a similarly increased risk for T2D diagnosed at ≥ 46 years in males (1.57-times) and females (1.46-times) than small alleles (S) with ≤ 7 R (3, 4, 5, 6, 7; as the reference; Fig. 3B). In contrast, L alleles conferred an opposite risk for T2D diagnosed at ≤ 45 years in males and females: while they conferred a 1.46-times increased risk in males, they decreased 25% the risk in females (Fig. 3A).

The associations of T2D with genotypes were greater than those observed with alleles (Online Resource 3); in males diagnosed at any age (Figs. 3D, E, F) and in women diagnosed at ≥ 46 years (Fig. 3E) the homozygous L/L genotypes conferred a much higher risk than heterozygous L/S genotypes. For example, the risk in males increased from an OR of 1.48 (95% CI 1.13–1.95; *p* = 0.005) when heterozygous to 2.26 (95% CI 1.41–3.64; *p* = 0.0007) when homozygous (Fig. 3F), indicating an additive effect with the number of alleles with ≥ 8R.

### Frequency of alleles and genotypes of SNP rs689 and its association with T2D

The frequency of alleles and genotypes of *rs689* was assessed in the main and replica studies (n = 4346). Allele A was significantly more frequent in cases than in controls (23.8% vs. 20.0%; *p* < 0.0001); although this difference was observed in females (22.6% vs. 20.2%; p < 0.05), it was markedly greater in males (25% vs. 19.6%; p < 0.001) (Fig. 2F). Notoriously, when the analysis was performed stratifying the sample by age of T2D presentation, the difference in men was statistically significant only in those diagnosed at ≤ 45 years (Fig. 2D), while the difference in women was only significant in those diagnosed at ≥ 46 years (Fig. 2E). Similar differences were found in the frequency of genotypes AT and AA between all cases and controls (Online Resource 4).

Logistic regression analysis demonstrated that allele A confers a significantly higher risk for T2D (OR 1.25; 95% CI 1.1–1.4; *p* < 0.0001), with a comparatively higher risk in males (OR 1.37; 95% CI 1.2–1.6; *p* < 0.001) than in females (OR 1.15; 95% CI 1–1.3; *p* = 0.043) (Fig. 3C). Likewise, as in the frequency comparison, the allele A confers a high risk for T2D only in men diagnosed early (OR 1.45; 95% CI 1.2–1.9, *p* < 0.001) and in women diagnosed late (OR 1.24; 95% CI 1–1.5; *p* = 0.032). (Figs. 3A, B).

For T2D diagnosed early, both genotypes (AT/AA) were found to confer a risk only in males, with an additive effect; the risk was much greater for AA (OR 2.2; 95% CI 1.2–4.1; *p* = 0.013) than AT (OR 1.53; 95% CI 1.2–2; *p* = 0.002) (Fig. 3D). For T2D diagnosed at ≥ 46 years (Fig. 3E), a higher risk was conferred only by AA in females (OR 2.44; 95% CI 1.4–4.2; *p* = 0.0015) and AT in males (OR 1.38; 95% CI 1–1.9; *p* = 0.036). Despite the similar differences across the studies in the allelic and genotypic frequencies, the risk conferred by allele A, or genotypes AT and AA, towards T2D diagnosis was observed mainly in males diagnosed at an earlier age in the two replica studies (Online Resources 5–10).

### Linkage disequilibrium analysis

**Fig. 4:**
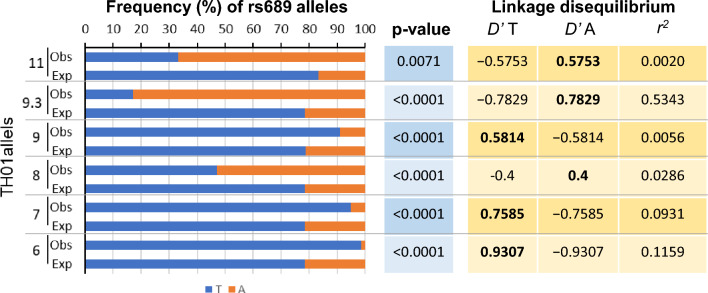
Linkage disequilibrium between *TH01* and SNP rs689 alleles. The analysis was performed only in the main case–control study (*n* = 1986; 3972 chromosomes). The graph shows the frequency (observed) with which each A and T allele of the rs689 SNP is inherited in conjunction with each of the *TH01* alleles in phased data. The expected frequency was calculated assuming that alleles A and T are not linked to alleles of *TH01*. The significance of the difference between the two frequencies was calculated with the chi-square test (*p* value). *D’* linkage disequilibrium statistic, *r*^2^ correlation between allelic values at two loci

Our analyses showed that *TH01* alleles with 3–7 repeats were in linkage disequilibrium with allele T of SNP rs689 (Fig. 4). Alleles 6 and 7 of *TH01* were inherited with allele T of SNP rs689 98.5% (*D’* = 0.9307) and 94.8% (*D’* = 0.7585) of the time. The differences in the frequency of haplotypes 6/T vs. 6/A and 7/T vs. 7/A completely departed from the expected random distribution if they were in linkage equilibrium (*p* < 0.00001). In contrast, *TH01* alleles with 8–11 repeats, except allele 9, were in partial linkage disequilibrium with allele A of SNP rs689. Allele 9.3 of *TH01* was highly linked and inherited with allele A for 83.0% of the time (*D’* = 0.7829). In contrast, alleles 8 and 11 were partially linked to allele A and segregated together for 52.9% and 66.7% of the time, respectively, and their respective *D’* values were lower (0.3882 and 0.5753). Similarly, the frequencies of haplotypes 9.3/A, 8/A, and 11/A were much more frequent than haplotypes with a T than if they were in equilibrium and randomly distributed (*p* < 0.01). Allele 9 was in linkage disequilibrium with allele T, and they were inherited together 90.1% of the time (*D’* = 0.5814).

### Multivariate logistic regression of *TH01* and SNP rs689

*TH01* and SNP rs689 alleles were both associated with T2D in the same groups as reported in univariate logistic regression models; the association remained significant for both loci in MLR models, which is explained by the partial linkage disequilibrium between some alleles of these two markers, suggesting that both loci contribute independently to T2D in the MLR model (Online Resource 11). In fact, the value of *R*^2^ increased from the first (*TH01*) to the second (rs689) block introduced in this model, supporting the additive contribution of the second marker, which could be indicative of a combined effect by which both loci contribute to T2D.

In males, both ≥ 8R haplotypes were associated with T2D, with haplotype ≥ 8R/T conferring a higher risk than haplotype ≥ 8R/A (Online Resource 12). This finding indicates a predominant effect of the microsatellite *TH01* over that of rs689. When explored individually, the T allele of the SNP rs689 confers protection, although, in females, the effect differed between age groups. In females, ≥ 8R/T was protective for T2D development at ≤ 45 years, while ≥ 8R/A had no effect; the opposite effect was observed among the groups diagnosed with T2D at ≥ 46 years of age.

### Association of insulin concentration with age and the alleles of *TH01* and rs689

The median fasting plasma insulin concentration (mIU/mL) was significantly higher among cases than in controls (9.5 [5.5–16.2] vs 6.8 [4.8–10]; *p* < 0.0001) (Online Resource 13). Interestingly, insulin concentrations were higher in cases diagnosed at ≤ 45 years than at ≥ 46 years; this difference was greater in males than in females. In fact, insulin concentrations decreased with age at T2D diagnosis (*r* = − 0.111; *p* < 0.0001), which was much greater in males (*r* = − 0.189; *p* < 0.0001) than in females (*r* = − 0.051; *p* > 0.05) and independent of allele type, suggesting the effect was likely related to age.

**Fig. 5:**
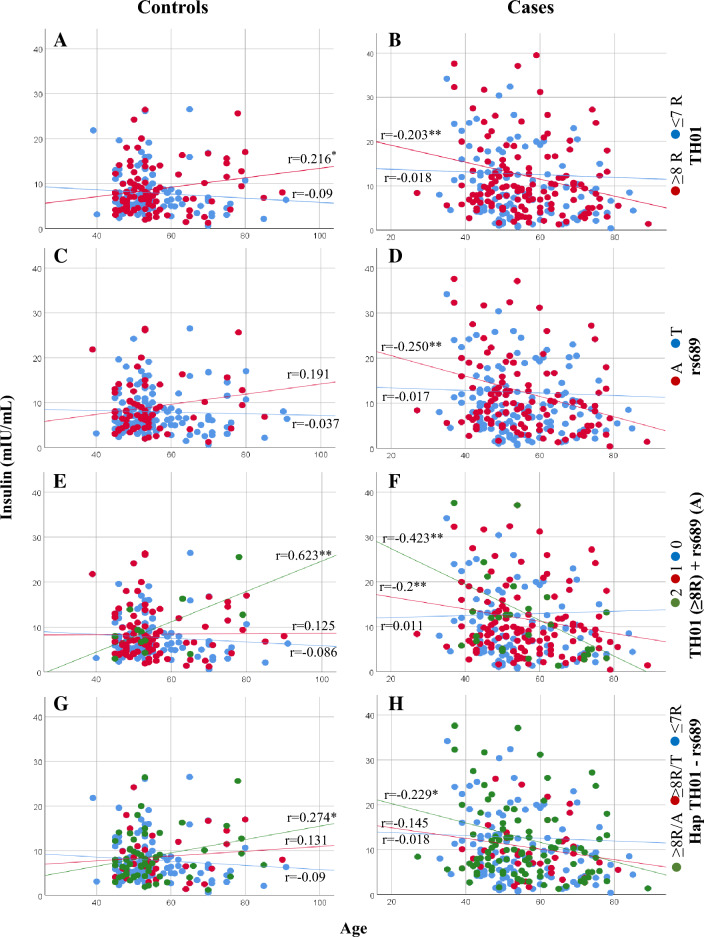
Correlation between fasting plasma insulin concentration and age of controls and cases in males according to *TH01* and rs689 alleles. **A**, **B** *TH01* alleles **C**, **D** SNP rs689 alleles **E**, **F** *TH01* ≥ 8R alleles + /or rs689 A allele and **G**, **H** Hap *TH01*—rs689. In panels **E**, **F**, the numbers 2 (green circle), 1 (red circle), and 0 (blue circle) represent *TH01* (≥ 8R) + rs689 A allele, *TH01* (≥ 8R) or rs689 A allele, and neither, respectively. *p < 0.05, **p < 0.01. *R* repeats, *SNP* single nucleotide polymorphism

**Fig. 6:**
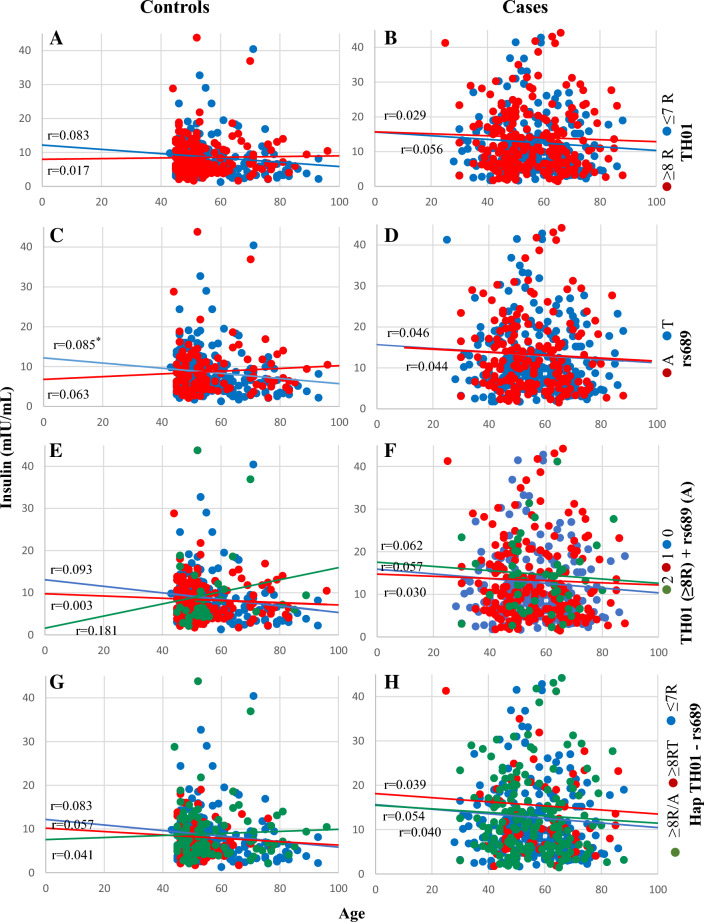
Correlation between fasting plasma insulin concentration and age of controls and cases in females according to TH01 and rs689 alleles. **A**, **B** *TH01* alleles **C**, **D** SNP rs689 alleles **E**, **F** *TH01* ≥ 8R alleles + /or rs689 A allele and **G**, **H** Hap *TH01*—rs689. In panels **E**, **F**, the numbers 2 (green circle), 1 (red circle), and 0 (blue circle) represent *TH01* (≥ 8R) + rs689 A allele, *TH01* (≥ 8R) or rs689 A allele, and neither, respectively. *p < 0.05, **p < 0.01. *R* repeats, *SNP* single nucleotide polymorphism

Insulin concentration significantly decreased with participant age for the entire main study population (*r* = − 0.046; *p* = 0.02). In males with T2D, insulin decreased significantly with age in those with alleles that had ≥ 8R of *TH01* (*r* = − 0.203; *p* = 0.009; Fig. 5B) or the rs689 allele A (*r* = − 0.250; *p* = 0.004; Fig. 5D). When both alleles were present, there was a significant increase in the negative correlation (*r* = − 0.423; *p* = 0.007; Fig. 5F), which occurred only with ≥ 8R/A and not with  ≥ 8R/T. In contrast, in males in the control group, an inverse correlation was observed for those with these alleles, whereby the fasting plasma insulin concentration increased with age (Fig. 5A–G). However, no significant correlations between insulin concentration and age were observed in females with T2D or in the control group for the alleles of the two markers (Fig. 6A–H).

## Discussion

Our results indicate an association between *TH01* microsatellite and the SNP rs689 with T2D and fasting plasma insulin concentrations. The degree of association varied with age at T2D diagnosis and sex. *TH01* alleles with ≥ 8R and the rs689 A allele conferred an increased risk of developing T2D at any age in males and at ≥ 46 years in females, and a protective (≥ 8R) or neutral (A) effect towards developing T2D at ≤ 45 years among females. *TH01* alleles with ≤ 7R have either an inverse (allele 6) or a neutral (allele 7) effect. Fasting plasma insulin decreased linearly with age in male cases who had alleles with ≥ 8R or the rs689 A allele but increased slightly in controls with those alleles. In contrast, fasting plasma insulin remained constant in male cases and controls who had alleles with ≤ 7R or rs689 T allele. Contrarily, the decrease of insulin concentration with age in females is not influenced by *TH01* or rs689. Linkage disequilibrium and multivariate analysis suggest that *TH01* is partially in linkage imbalance with rs689 and that the risk or protection conferred by both loci for T2D appear to be independent of each other, even though there may be an additive effect.

Although inconsistent, most studies indicate that class I alleles stimulate the *INS* gene 1.5- to 3 times more than class III alleles [1, 5, 8, 9, 32–35]. While Le Stunff et al. noted an association between T/T genotypes (VNTR I/I) with a higher fasting plasma insulin level [32], other studies have found greater stimulation of allele III for insulin gene expression in in vitro experiments [6] or equal to allele I for plasma insulin secretion [7]. Allele A of rs689, linked with allele III, influences alternative splicing of intron 1 of *INS* through differential recognition of its 3′ splice site, resulting in an increased production of mature transcripts and more proinsulin in culture supernatants than transcripts from allele T [7].

The influence of VNTR on gene activity or plasma insulin secretion in T2D remains unknown. However, studies have demonstrated the involvement of VNTR in *INS* expression, a mechanism that could be altered or affected in T2D in some populations [2, 7]. Our results with SNP rs689 support this hypothesis, as insulin concentration significantly decreased with age in male participants with T2D who were positive for allele A (class III), while it remained constant in those positive for allele T (class I).

The association of ≥ 8R with a slight increase in fasting plasma insulin concentrations with age in controls suggests a probable involvement of *TH01* in regulating *INS* expression. However, because this association was also observed with allele A of rs689, the association between *TH01* microsatellite alleles and fasting plasma insulin levels could be due to a linkage effect with allele A. In cases with these alleles or with the ≥ 8R/A haplotype, fasting plasma insulin levels decreased with age, which could suggest that the regulation of *INS* expression could be progressively affected by age in the presence of class III (of *VNTR INS*) and/or ≥ 8R (of *TH01*) alleles. Because allele 6, which is completely linked to allele T of rs689, was found to be a protector for T2D and its frequency increased with age, it could have an inverse effect compared with the ≥ 8R/A haplotype on *INS* gene expression during aging.

Alleles with ≥ 8R are protective for T2D development at ≤ 45 years and confer a greater risk at a later age in females, indicating the crucial role of estrogen in this association. Epidemiological studies have demonstrated that estrogens are protective against T2D. First, hormone therapy has been shown to reduce the incidence of diabetes by 35% in postmenopausal females with coronary heart disease [36], and secondly, women with early menopause have a higher risk of T2D [37]. There is evidence that estrogen represses the expression of insulin mRNA in pancreatic β-cells through indirect genomic signaling [38] and can stimulate the degradation of misfolded proinsulin, thereby protecting the production of insulin and delaying the onset of diabetes [39]. Our analysis is limited by including only Mexican participants and, therefore, not necessarily generalizable to other populations.

In conclusion, insulin increases with age in males when *TH01* alleles with ≥ 8R or allele A of rs689 are present, suggesting an involvement in a mechanism that maintains insulin synthesis in individuals without T2D. In contrast, this is somehow disabled in patients with T2D, thus warranting further research.

### Supplementary Information

Below is the link to the electronic supplementary material.

Supplementary file1 (DOCX 26 KB)

Supplementary file2 (DOCX 185 KB)

## Data Availability

The datasets generated during and/or analyzed during the current study are available from the corresponding author on reasonable request.
