# Supplementary material for: Association of tyrosine hydroxylase 01 (TH01) microsatellite and insulin gene (INS) variable number of tandem repeat (VNTR) with type 2 diabetes and fasting insulin secretion in Mexican population
Source: J Endocrinol Invest. 2023 Aug 25;47(3):571–83. doi: 10.1007/s40618-023-02175-4 (PMC10904573; doi:10.1007/s40618-023-02175-4)
Supplement: Supplementary file 1 — Supplementary file1 (DOCX 26 KB) [file 40618_2023_2175_MOESM1_ESM.docx]

**Association of tyrosine hydroxylase *01* (*TH01*) microsatellite and insulin gene (*INS)* variable number of tandem repeat *(*VNTR) with type 2 diabetes and fasting insulin secretion in Mexican population.**

Jaime Berumen, Lorena Orozco, Héctor Gallardo-Rincón, Eligia Juárez-Torres, Elizabeth Barrera, Humberto García-Ortiz, Miguel Cruz-López, Rosa Elba Benuto, Espiridión Ramos-Martinez, Melissa Marin-Madina, Anabel Alvarado-Silva, Adán Valladares-Salgado, José de Jesús Peralta-Romero, Luis Alberto Martinez-Juarez, Alejandra Montoya, Diego-Abelardo Alvarez-Hernández, Jesús Alegre-Diaz, Pablo Kuri-Morales, Roberto Tapia-Conyer

**Corresponding author**

**Name:** Jaime Berumen

**Address:** Unidad de Investigación en Medicina Experimental, Facultad de Medicina Universidad Nacional Autónoma de México, CDMX Mexico

**Telephone:** +52 1 55 1850-4023

**Email:** jaimeberumen47@gmail.com

**Name:** Héctor Gallardo-Rincón

**Address:** Centro Universitario de Ciencias de la Salud, Universidad de Guadalajara, Sierra Mojada 950, Guadalajara 44340, Jalisco, Mexico

**Telephone:** +52 1 55 4181 8180

**Email:** hgallardo@fundacioncarlosslim.org

**Online Resource 1**

**Supplementary Text**

*Clinical information*

Main case–control study: weight, height, waist and hip circumference, and parental history of T2D was collected during the initial interview. For fasting glucose measurements and DNA extraction, 10 mL of intravenous blood was collected; all participants were assessed for *TH01* and rs689 markers.

Replica case–control study: Clinical history and anthropometric and biochemical measurements were recorded for all study participants.

Replica cross-sectional study: The participants were surveyed to assess T2D risk factors, and anthropometric measurements were collected. A blood sample was drawn to measure glycated hemoglobin (A1c) and assess DNA polymorphisms; individuals with A1c ≥ 6.5 were considered diabetic, and those with A1c < 6.5 were considered non-diabetic. The SNP, rs689 was genotyped in all people with T2D (*n* = 104), those with pre-diabetes (*n* = 529; A1c ≥ 5.7 to < 6.5), and controls aged ≥ 40 years (*n* = 539; A1c < 5.7). Those with pre-diabetes and controls were included in the non-diabetic group (*n* = 1068).

TH01 *and rs689 SNP genotyping*

Only DNA samples of the main study were genotyped for *TH01*, and all samples including those of the replica studies were genotyped for SNP rs689. The *TH01* microsatellite was assessed by polymerase chain reaction (PCR) using a fluorescence-labelled primer (5′-GTGGGCTGAAAAGCTCCCGATTAT-3′) and an unlabeled primer (5′-ATTCAAAGGGTATCTGGGCTCTGG-3′). Subsequently, alleles were identified by number of repeats (R) identified during capillary electrophoresis using the SeqStudio Genetic Analyzer (ThermoFisher Scientific, Waltham, MA, USA). Genotyping of the rs689 SNP was performed using the allelic discrimination assay-by-design TaqMan^®^ method (C_1223317_10) on 384-well plates analyzed on the QuantStudio™ 12 K Flex Real-Time PCR System (ThermoFisher Scientific). The genotypes were analyzed using Genotyper™ software v1.3 (ThermoFisher Scientific).

*Statistical analysis*

Baseline characteristics and insulin concentration were summarized using mean and standard deviation (SD) or median and interquartile range (25%–75%). To assess the statistical significance of intergroup differences, the Student’s *t-*test was used for mean values, and the Mann–Whitney *U* test for median values. We investigated whether the frequency of genotypes was distributed according to the Hardy–Weinberg law based on the allelic frequency and the formula: (*a* + *b*)^2^ = *a*^2^ + 2*ab* + *b*^2^, where *a* and *b* are the allelic frequencies in the control group. The fasting plasma insulin concentration trend was analyzed according to participant age; significance was calculated using the Pearson correlation test.

The analysis of both DNA polymorphism variables was stratified by sex and age at T2D diagnosis. For these subgroups, cases (≤ 45 and ≥ 46 years) and controls (≤ 54 and ≥ 55 years) were categorized by their median age at diagnosis or age at enrollment, respectively. The risk conferred by each factor (explanatory variables) was calculated by comparing the cases and controls using univariate logistic regression models. The reference indicators of the explanatory variables, *TH01* and rs689, were *TH01* alleles with ≤ 7R and rs689 = T. The association was expressed as the OR and 95% CI, and the contribution to the variability of T2D was expressed as Nagelkerke’s R^2^. In the multivariate model, the factors were included successively in the model in different blocks. The contribution of each factor to the model was assessed by the increase of *R*^2^ and the decrease in the −2 log likelihood ratio value from one block to the next; the Omnibus test was used to determine statistical significance between the successive blocks.

For the analysis, the presence or absence of T2D was considered the dependent variable, and the values of the alleles or genotypes were considered the explanatory variables. In addition, for MLR models, the value of the total *r*^2^ obtained at the end of the model was introduced for the power calculation.

No corrections for relatedness were made because the median of the global genomic relationship among individuals was −0.0054 IQR [−0.0011–0.0000668]. The global genomic relationship between individuals in the studied sample was explored with 69 SNPs (medRxiv preprint doi: https://doi.org/10.1101/2022.10.27.22281587) using the GLUP algorithm ([Genomic Best Linear Unbiased Predictors Analysis Using Bins](qthelp://org.sphinx.svsmanual.8.9.1/doc/svsmanual/mixedModelMethods/binned_gblup.html#binnedgblup)) with the Golden Helix software.
