# Supplementary material for: Association of tyrosine hydroxylase 01 (TH01) microsatellite and insulin gene (INS) variable number of tandem repeat (VNTR) with type 2 diabetes and fasting insulin secretion in Mexican population
Source: J Endocrinol Invest. 2023 Aug 25;47(3):571–83. doi: 10.1007/s40618-023-02175-4 (PMC10904573; doi:10.1007/s40618-023-02175-4)
Supplement: Supplementary file 2 — Supplementary file2 (DOCX 185 KB) [file 40618_2023_2175_MOESM2_ESM.docx]

Supplementary Material

Association of *TH01* microsatellite and *INS* VNTR with type 2 diabetes and fasting insulin secretion

# Jaime Berumen, Lorena Orozco, Héctor Gallardo-Rincón, Eligia Juárez-Torres, Elizabeth Barrera^,^ Miguel Cruz-López, Rosa Elba Benuto, Espiridión Ramos-Martinez, Melissa Marin- Madina, Anabel Alvarado-Silva, Adán Valladares-Salgado, José de Jesús Peralta-Romero, Humberto García-Ortiz, Luis Alberto Martinez-Juarez, Alejandra Montoya, Diego-Abelardo Alvarez-Hernández, Jesús Alegre-Diaz, Pablo Kuri-Morales, Roberto Tapia-Conyer

**Corresponding author Name:** Jaime Berumen

# **Address:** Unidad de Investigación en Medicina Experimental, Facultad de Medicina Universidad Nacional Autónoma de México, CDMX Mexico

**Telephone:** +52 1 55 1850-4023

**Email:** [jaimeberumen47@gmail.com](mailto:jaimeberumen47@gmail.com)

# Héctor Gallardo-Rincón

Centro Universitario de Ciencias de la Salud, Universidad de Guadalajara, Sierra Mojada 950, Guadalajara 44340, Jalisco, Mexico

**Online Resource 2.** Comparison of frequency of *TH01* alleles with a minor allele frequency ≥ 0.05 between controls and cases (*n =* 3972).

| **Allele** |  |  | **Allelic frequency** |  |  |  |
| --- | --- | --- | --- | --- | --- | --- |
|  | **Controls**  **≤ 54 years** | **Cases Dx**  **≤ 45 years** | **Controls**  **≥ 55 years** | **Cases Dx**  **≥ 46 years** | **All controls** | **All cases** |
| **Female** | *n =* 462 | *n =* 536 | *n =* 608 | *n =* 506 | *n =* 1070 | *n =* 1048 |
| 6 | 31.2 (144) | 32.3 (173) | **38.2 (232)^‡^** | 29.4 (149) | **35.1 (376)^†^** | 30.9 (324) |
| 7 | 32.7 (151) | 37.1 (199) | 38.2 (232) | 38.9 (197) | 35.8 (383) | 38.0 (398) |
| 8 | 6.1 (28) | 4.5 (24) | 3.3 (20) | 4.9 (25) | 4.5 (48) | 4.7 (49) |
| 9 | **9.5 (44)^‡^** | 4.7 (25) | 4.6 (28) | 4.9 (25) | 6.7 (72) | 5.0 (52) |
| 9.3 | 20.1 (93) | 20.3 (109) | 15.8 (96) | **21.1 (107)^†^** | 17.7 (189) | 20.6 (216) |
| **Male** | *n =* 464 | *n =* 470 | *n =* 462 | *n =* 454 | *n =* 926 | *n =* 928 |
| 6 | 36.2 (168) | 31.1 (146) | **40.5 (187)^¶^** | 27.8 (126) | **38.3 (355)^¶^** | 29.4 (273) |
| 7 | 38.8 (180) | 36.4 (171) | 35.3 (163) | 38.5 (175) | 37.0 (343) | 37.4 (347) |
| 8 | 3.4 (16) | 4.9 (23) | 3.0 (14) | **6.6 (30)^†^** | 3.2 (30) | **5.8 (54)^‡^** |
| 9 | 3.7 (17) | 5.1 (24) | 4.5 (21) | 6.2 (28) | 4.1 (38) | 5.7 (53) |
| 9.3 | 17.5 (81) | **22.3 (105)^*^** | 16.5 (76) | **20.7 (94)^*^** | 17.0 (157) | **21.4 (199)^†^** |
| **Both sexes** | *n =* 926 | *n =* 1006 | *n =* 1070 | *n =* 960 | *n =* 1996 | *n =* 1976 |
| 6 | 33.7 (312) | 31.7 (319) | **39.2 (419)^¶^** | 28.6 (275) | **36.6 (730)^¶^** | 30.2 (597) |
| 7 | 35.7 (331) | 36.8 (370) | 36.9 (395) | 38.8 (372) | 36.4 (726) | 37.7 (745) |
| 8 | 4.8 (44) | 4.7 (47) | 3.2 (34) | **5.7 (55)^‡^** | 3.9 (78) | **5.2 (103)^†^** |
| 9 | 6.6 (61) | 4.9 (49) | 4.6 (49) | 5.5 (53) | 5.5 (110) | 5.3 (105) |
| 9.3 | 18.8 (174) | 21.3 (214) | 16.1 (172) | **20.9 (201)^‡^** | 17.3 (346) | **21.0 (415)^‡^** |

*Dx* diagnosis, *R* repeats.

Cases diagnosed with type 2 diabetes at ≤ 45 years were compared with controls aged ≤ 54 years and cases diagnosed with type 2 diabetes at ≥ 46 years were compared with controls aged ≥ 55 years.

^*^*p* < 0.1; ^†^*p* < 0.05, ^‡^*p* < 0.01, ^§^*p* < 0.001, ^¶^*p* < 0.0001

The Pearson chi square test was used to assess the statistical significance; statistically significant (*p* < 0.05) and *p* < 0.1 values are highlighted in bold.

**Online Resource 3.** Association of *TH01* genotypes with T2D stratified by age at T2D diagnosis and sex (*n =* 1986)^a^.

**Univariate logistic regression models**

**No. repeats No. risk alleles Diagnosed at ≤ 45 years Diagnosed at ≥ 46 years All cases**

|  | | **OR (95% CI)** | ***p value*** | **OR (95% CI)** | ***p value*** | **OR (95% CI)** | ***p value*** |
| --- | --- | --- | --- | --- | --- | --- | --- |
| **Female** |  | *n =* 268 |  | *n =* 253 |  | *n =* 524 |  |
|  | 0 | 1 |  | 1 |  | 1 |  |
| 6 | 1 | 1.17 (0.81–1.7) | 0.4 | 0.83 (0.58–1.19) | 0.3 | 0.99 (0.77–1.28) | 0.93 |
|  | 2 | 0.97 (0.51–1.83) | 0.91 | 0.4 (0.23–0.7) | **0.0015** | 0.57 (0.38–0.86) | **0.0074** |
| 8 | 0 | 1 |  | 1 |  | 1 |  |
|  | ≥ 1 | 0.67 (0.37–1.23) | 0.19 | 1.43 (0.75–2.7) | 0.27 | 0.97 (0.63–1.51) | 0.9 |
| 9 | 0 | 1 |  | 1 |  | 1 |  |
|  | ≥ 1 | 0.46 (0.27–0.79) | **0.0044** | 1.03 (0.58–1.83) | 0.91 | 0.72 (0.49–1.05) | 0.088 |
|  | 0 | 1 |  | 1 |  | 1 |  |
| 9.3 | 1 | 1.15 (0.78–1.7) | 0.47 | 1.7 (1.17–2.48) | **0.0052** | 1.41 (1.08–1.85) | **0.012** |
|  | 2 | 0.75 (0.33–1.73) | 0.5 | 1.18 (0.5–2.79) | 0.71 | 0.95 (0.52–1.72) | 0.86 |
| **Male** |  | *n =* 235 |  | *n =* 227 |  | *n =* 464 |  |

|  | 0 | 1 |  | 1 |  | 1 |  |
| --- | --- | --- | --- | --- | --- | --- | --- |
| 6 | 1 | 0.8 (0.54–1.18) | 0.27 | 0.59 (0.4–0.88) | **0.0094** | 0.69 (0.52–0.91) | **0.0091** |
|  | 2 | 0.66 (0.37–1.16) | 0.15 | 0.3 (0.15–0.56) | **0.0002** | 0.46 (0.3–0.7) | **0.0003** |
| 8 | 0 | 1 |  | 1 |  | 1 |  |
|  | ≥ 1 | 1.39 (0.71–2.73) | 0.33 | 2.57 (1.27–5.19) | **0.0086** | 1.92 (1.19–3.1) | **0.0078** |
| 9 | 0 | 1 |  | 1 |  | 1 |  |
|  | ≥ 1 | 1.44 (0.75–2.75) | 0.27 | 1.44 (0.77–2.69) | 0.25 | 1.46 (0.94–2.29) | 0.095 |
|  | 0 | 1 |  | 1 |  | 1 |  |
| 9.3 | 1 | 1.35 (0.91–2.01) | 0.14 | 1.55 (1.04–2.32) | **0.033** | 1.44 (1.08–1.91) | **0.012** |
|  | 2 | 1.81 (0.73–4.49) | 0.2 | 1.02 (0.36–2.88) | 0.97 | 1.41 (0.72–2.78) | 0.32 |
| **Both sexes** |  |  |  |  |  |  |  |
|  | 0 | 1 |  | 1 |  | 1 |  |
| 6 | 1 | 0.98 (0.75–1.29) | 0.9 | 0.72 (0.55–0.93) | **0.013** | 0.84 (0.69–1.01) | **0.065** |
|  | 2 | 0.78 (0.51–1.19) | 0.25 | 0.35 (0.23–0.54) | **< 0.0001** | 0.51 (0.38–0.69) | **< 0.0001** |
| 8 | 0 | 1 |  | 1 |  | 1 |  |
|  | ≥ 1 | 0.94 (0.6–1.46) | 0.77 | 1.89 (1.19–3.01) | **0.0074** | 1.33 (0.97–1.83) | 0.078 |

| 0  9 | | 1 | 1 | | 1 | | |
| --- | --- | --- | --- | --- | --- | --- | --- |
|  | ≥ 1 | 0.74 (0.49–1.1) | 0.14 | 1.21 (0.79–1.84) | 0.38 | 0.97 (0.73–1.29) | 0.83 |
|  | 0 | 1 |  | 1 |  | 1 |  |
| 9.3 | 1 | 1.25 (0.94–1.64) | 0.12 | 1.64 (1.24–2.15) | **0.0004** | 1.42 (1.17–1.73) | **0.0004** |
|  | 2 | 1.13 (0.62–2.08) | 0.69 | 1.1 (0.57–2.14) | 0.78 | 1.13 (0.72–1.76) | 0.6 |

*CI* confidence interval, *OR* odds ratio, *T2D* type 2 diabetes.

^a^ A total of 998 controls were included, 535 females and 463 males.

Cases diagnosed with T2D at ≤ 45 years were compared with controls aged ≤ 54 years and cases diagnosed with T2D at ≥ 46 years were compared with controls aged ≥ 55 years.

The Wald test was used to assess the statistical significance using the Enter method; statistically significant (*p* < 0.05) and *p* < 0.1 values are highlighted in bold.

The power (1−β error probability) > 0.99 in all groups with p < 0.05.

# **Online Resource 4** Comparison of frequency of rs689 genotypes between controls and cases of type 2 diabetes stratified by age at type 2 diabetes diagnosis and sex (*n =* 4341).

| **Genotypic frequency % (*n*)** | | | | | | |
| --- | --- | --- | --- | --- | --- | --- |
| **Genotypes** | **Controls ≤**  **54 years** | **Cases Dx ≤ 45**  **years** | **Controls ≥ 55**  **years** | **Cases Dx ≥**  **46 years** | **All controls** | **All cases** |
| **Female** | *n =* 1030 | *n =* 433 | *n =* 717 | *n =* 457 | *n =* 1747 | *n =* 890 |
| **TT** | 63.5 (654) | 62.1 (269) | 63.9 (458) | **60.8 (278)** | 63.7 (1112) | **61.5 (547)** |
| **AT** | 31.7 (327) | 31.9 (138) | 32.9 (236) | **31.7 (145)** | 32.2 (563) | **31.8 (283)** |
| **AA** | 4.8 (49) | 6.0 (26) | 3.2 (23) | **7.4 (34)^‡^** | 4.1 (72) | **6.7 (60)^*^** |
| **AA/AT** | 36.5 (376) | 37.9 (164) | 36.1 (259) | 39.2 (179) | 36.3 (635) | 38.5 (343) |
| **Male** | *n =* 519 | *n =* 412 | *n =* 394 | *n =* 379 | *n =* 913 | *n =* 791 |
| **TT** | 64.9 (337) | **53.6 (221)** | 64.7 (255) | 57.5 (218) | 64.8 (592) | **55.5 (439)** |
| **AT** | 31.6 (164) | **40.0 (165)** | 30.7 (121) | 37.7 (143) | 31.2 (285) | **38.9 (308)** |
| **AA** | 3.5 (18) | **6.3 (26)^‡^** | 4.6 (18) | 4.7 (18) | 3.9 (36) | **5.6 (44) ^‡^** |
| **AA/AT** | 35.1 (182) | 46.4 (191) | 35.3 (139) | 42.5 (161) | 35.2 (321) | 44.5 (352) |
| **Both sexes** | *n =* 1549 | *n =* 845 | *n =* 1111 | *n =* 836 | *n =* 2660 | *n =* 1681 |
| **TT** | 64.0 (991) | **58.0 (490)** | 64.2 (713) | **59.3 (496)** | 64.1 (1704) | **58.7 (986)** |
| **AT** | 31.7 (491) | **35.9 (303)** | 32.1 (357) | **34.4 (288)** | 31.9 (848) | **35.2 (591)** |
| **AA** | 4.3 (67) | **6.2 (52)^‡^** | 3.7 (41) | **6.2 (52)^†^** | 4.1 (108) | **6.2 (104)^‡^** |
| **AA/AT** | 36.0 (558) | 42.0 (355) | 35.8 (398) | 40.7 (340) | 35.9 (956) | 41.3 (695) |

Statistically significant values are highlighted in bold (Pearson chi square test, 2×3 table).

^*^*p* < 0.1; ^†^*p* < 0.05, ^‡^*p* < 0.01

# **Online Resource 5** Frequency of rs689 alleles (*n* = 3962) and genotypes (*n* = 1981) in the main case–control study stratified by sex and age of type 2 diabetes diagnosis.

|  | **Controls**  **≤ 54 years** | **Cases Dx**  **≤ 45 years** | **Controls**  **≥ 55 years** | **Cases Dx**  **≥ 46 years** | **All controls** | **All cases** |
| --- | --- | --- | --- | --- | --- | --- |
| **Alleles** |  |  | **Allelic frequency % (*n*)** | |  |  |
| **Female** | *n =* 462 | *n =* 532 | *n =* 608 | *n =* 504 | *n =* 1070 | *n =* 1042 |
| **T** | 78.1 (361) | 77.8 (414) | 81.7 (497) | 76.4 (385) | 80.2 (858) | 77.3 (805) |
| **A** | 21.9 (101) | 22.2 (118) | 18.3 (111) | **23.6 (119)^†^** | 19.8 (212) | 22.7 (237) |
| **Male** | *n =* 462 | *n =* 468 | *n =* 462 | *n =* 454 | *n =* 924 | *n =* 926 |
| **T** | 80.1 (370) | 74.4 (348) | 82.9 (383) | 76.4 (347) | 81.5 (753) | 75.3 (697) |
| **A** | 19.9 (92) | **25.6 (120)^†^** | 17.1 (79) | **23.6 (107)^†^** | 18.5 (171) | **24.7 (229)**^‡^ |
| **Both sexes** | *n =* 924 | *n =* 1000 | *n =* 1070 | *n =* 958 | *n =* 1994 | *n =* 1968 |
| **T** | 79.1 (731) | 76.2 (762) | 82.2 (880) | 76.4 (732) | 80.8 (1611) | 76.3 (1502) |
| **A** | 20.9 (193) | 23.8 (238) | 17.8 (190) | **23.6 (226)^§^** | 19.2 (383) | **23.7 (466)**^‡^ |
| **Genotypes** |  |  | **Genotypic frequency % (*n*)** | | |  |
| **Female** | *n =* 231 | *n =* 268 | *n =* 304 | *n =* 253 | *n =* 535 | *n =* 521 |
| **TT** | 62.8 (145) | 60.4 (162) | 67.4 (205) | 59.3 (150) | 65.4 (350) | 60.1 (315) |
| **AT** | 30.7 (71) | 33.6 (90) | 28.6 (87) | 33.6 (85) | 29.5 (158) | 33.4 (175) |
| **AA** | 6.5 (15) | 5.2 (14) | 3.9 (12) | 6.7 (17) | 5 (27) | 5.9 (31) |
| **AA/AT** | 37.2 (86) | 39.1 (104) | 32.6 (99) | 40.5 (102) | 34.6 (185) | **39.5 (206)^*^** |

| **Male** | *n =* 232 | *n =* 235 | *n =* 231 | *n =* 227 | *n =* 462 | *n =* 463 |
| --- | --- | --- | --- | --- | --- | --- |
| **TT** | 64.2 (149) | 54.9 (129) | 70.6 (163) | **57.3 (130)** | 67.4 (312) | **55.8 (259)** |
| **AT** | 31 (72) | 38.3 (90) | 24.7 (57) | **38.3 (87)** | 27.9 (129) | **38.6 (179)** |
| **AA** | 4.3 (10) | 6.4 (15) | 4.8 (11) | **4.4 (10)**^‡^ | 4.5 (21) | **5.4 (25)**^‡^ |
| **AA/AT** | 35.5 (82) | **44.9 (105)^†^** | 29.4 (68) | **42.7 (97)**^‡^ | 32.5 (150) | **44.1 (204)^§^** |
| **Both sexes** | *n =* 463 | *n =* 503 | *n =* 535 | *n =* 480 | *n =* 997 | *n =* 984 |
| **TT** | 63.5 (294) | 57.9 (291) | 68.8 (368) | **58.3 (280)** | 66.3 (662) | **58.1 (574)** |
| **AT** | 30.9 (143) | 35.8 (180) | 26.9 (144) | **35.8 (172)** | 28.8 (287) | **35.8 (354)** |
| **AA** | 5.4 (25) | 5.8 (29) | 4.3 (23) | **5.6 (27)**^‡^ | 4.8 (48) | **5.7 (56)^§^** |
| **AA/AT** | 36.4 (168) | **41.8 (209)^*^** | 31.2 (167) | **41.5 (199)^§^** | 33.6 (335) | **41.7 (410)^§^** |

Cases diagnosed with type 2 diabetes at ≤ 45 years were compared with controls aged ≤ 54 years and cases diagnosed with type 2 diabetes at ≥ 46 years were compared with controls aged ≥ 55 years.

Statistically significant values are highlighted in bold [Pearson chi square test, 2×3 (TT vs. AT vs. AA) or 2×2 (TT vs. AA/AT) tables].

^*^*p* < 0.1; ^†^*p* < 0.05, ^‡^*p* < 0.01, ^§^*p* < 0.001

# **Online Resource 6** Association of single nucleotide polymorphism rs689 alleles (*n* = 3952)^a^ and genotypes (*n* = 1976)^b^ with type 2 diabetes in the main case–control study stratified by age at type 2 diabetes diagnosis and sex.

| **Univariate logistic regression models** | | | | | | |
| --- | --- | --- | --- | --- | --- | --- |
|  | **T2D Dx ≤ 45 years** | | **T2D Dx ≥ 46 years** | | **All cases** | |
|  | **OR (95% CI)** | ***p value*** | **OR (95% CI)** | ***p value*** | **OR (95% CI)** | ***p value*** |
| **rs689 alleles** |  |  |  |  |  |  |
| **Female** | *n =* 462/532 |  | *n =* 608/504 |  | *n =* 1070/1042 | |
| **T** | 1 |  | 1 |  | 1 |  |
| **A** | 1.02 (0.75-1.38) | 0.90 | **1.38 (1.03-1.85)** | **0.029** | 1.19 (0.97-1.47) | 0.10 |
| **Male** | *n =* 462/468 |  | *n =* 462/454 |  | *n =* 924/926 | |
| **T** | 1 |  | 1 |  | 1 |  |
| **A** | **1.39 (1.02-1.89)** | **0.038** | **1.49 (1.08-2.07)** | **0.015** | **1.45 (1.16-1.81)** | **0.0012** |
| **Both sexes** | *n =* 924/1000 |  | *n =* 1070/958 |  | *n =* 1994/1968 | |
| **T** | 1 |  | 1 |  | 1 |  |
| **A** | 1.18 (0.95-1.47) | 0.13 | **1.43 (1.15-1.78)** | **0.0012** | **1.31 (1.12-1.52)** | **0.0006** |
| **No. risk alleles^c^** | | | | | | |
| **Female** | *n =* 266 |  | *n =* 252 |  | *n =* 518 | |
| **0** | 1 |  | 1 |  | 1 |  |
| **1** | 1.13 (0.77-1.66) | 0.52 | 1.34 (0.93-1.92) | 0.12 | 1.23 (0.95-1.6) | 0.12 |
| **2** | 0.84 (0.39-1.79) | 0.64 | 1.94 (0.9-4.17) | 0.092 | 1.28 (0.74-2.18) | 0.37 |
| **Male** | *n =* 234 |  | *n =* 227 |  | *n =* 461 | |
| **0** | 1 |  | 1 |  | 1 |  |
| **1** | 1.44 (0.98-2.13) | 0.064 | **1.91 (1.28-2.87)** | **0.0017** | **1.67 (1.26-2.21)** | **0.0003** |

| **2** | 1.73 (0.75-3.99) | 0.20 | 1.14 (0.47-2.77) | 0.77 | 1.43 (0.78-2.62) | 0.24 |
| --- | --- | --- | --- | --- | --- | --- |
| **Both sexes** | *n =* 500 |  | *n =* 479 |  | *n =* 979 | |
| **0** | 1 |  | 1 |  | 1 |  |
| **1** | 1.27 (0.97-1.67) | 0.084 | **1.57 (1.2-2.06)** | **0.0011** | **1.42 (1.17-1.72)** | **0.0003** |
| **2** | 1.17 (0.67-2.05) | 0.58 | 1.54 (0.87-2.75) | 0.14 | 1.35 (0.9-2.01) | 0.15 |

*CI* confidence interval, *Dx* diagnosis, *n* controls/cases, *OR* odds ratio Statistically significant values are highlighted in bold (Enter method, Wald test).

^a^ Included 1994 controls (chromosomes) (female, *n* = 1070; male, *n* = 924). Cases diagnosed at ≤ 45 years were compared with controls ≤ 54 years old (*n =*

924) and cases diagnosed at ≥ 46 years were compared with controls ≥ 55 years old (*n =* 1070).

^b^ Included 997 controls (female, *n* = 535; male, *n* = 462). Cases diagnosed at ≤ 45 years were compared with controls ≤ 54 years old (*n =* 462) and cases diagnosed at ≥ 46 years were compared with controls ≥ 55 years old (*n =* 535).

^c^ 0 = TT, 1 = AT, and 2 = AA.

The power (1−β error probability) > 0.99 in all groups with p < 0.05.

# **Online Resource 7** Frequency of rs689 alleles (*n =* 2376) and genotypes (*n =* 1188) in the replica case–control study stratified by age at type 2 diabetes diagnosis and sex.

|  | **Controls**  **≤ 54 years** | **Cases Dx**  **≤ 45 years** | **Controls**  **≥ 55 years** | **Cases Dx**  **≥ 46 years** | **All controls** | **All cases** |
| --- | --- | --- | --- | --- | --- | --- |
| **Alleles** |  |  | **Allelic frequency % (*n*)** | |  |  |
| **Female** | *n =* 486 | *n =* 296 | *n =* 360 | *n =* 294 | *n =* 846 | *n =* 594 |
| **T** | 77.6 (377) | 77.4 (229) | 76.9 (277) | 75.5 (222) | 77.3 (654) | 76.6 (455) |
| **A** | 22.4 (109) | 22.6 (67) | 23.1 (83) | 24.5 (72) | 22.7 (192) | 23.4 (139) |
| **Male** | *n =* 158 | *n =* 330 | *n =* 186 | *n =* 260 | *n =* 344 | *n =* 592 |
| **T** | 80.4 (127) | 73.3 (242) | 76.3 (142) | 77.3 (201) | 78.2 (269) | 75 (444) |
| **A** | **19.6 (31)** | **26.7 (88)^*^** | 23.7 (44) | 22.7 (59) | 21.8 (75) | 25.0 (148) |
| **Both sexes** | *n =* 644 | *n =* 626 | *n =* 546 | *n =* 554 | *n =* 1190 | *n =* 1186 |
| **T** | 78.3 (504) | 75.2 (471) | 76.7 (419) | 76.4 (423) | 77.6 (923) | 75.8 (899) |
| **A** | 21.7 (140) | 24.8 (155) | 23.3 (127) | 23.6 (131) | 22.4 (267) | 24.2 (287) |
| **Genotypes** |  |  | **Genotypic frequency % (*n*)** | | |  |
| **Female** | *n =* 243 | *n =* 144 | *n =* 180 | *n =* 153 | *n =* 423 | *n =* 297 |
| **TT** | 61.3 (149) | 63.9 (92) | 56.7 (102) | **59.5 (91)** | 59.3 (251) | **61.6 (183)** |
| **AT** | 32.5 (79) | 28.5 (41) | 40.6 (73) | **31.4 (48)** | 35.9 (152) | **30.0 (89)** |
| **AA** | 6.2 (15) | 7.6 (11) | 2.8 (5) | **9.2 (14)^†^** | 4.7 (20) | **8.4 (25)^*^** |
| **AA/AT** | 38.7 (94) | 36.1 (52) | 43.3 (78) | 40.5 (62) | 40.7 (172) | 38.4 (114) |

| **Male** | *n =* 79 | *n =* 164 | *n =* 93 | *n =* 132 | *n =* 172 | *n =* 296 |
| --- | --- | --- | --- | --- | --- | --- |
| **TT** | 65.8 (52) | 52.4 (86) | 54.8 (51) | 58.3 (77) | 59.9 (103) | 55.1 (163) |
| **AT** | 29.1 (23) | 42.1 (69) | 43.0 (40) | 37.1 (49) | 36.6 (63) | 39.9 (118) |
| **AA** | 5.1 (4) | 5.5 (9) | 2.2 (2) | 4.5 (6) | 3.5 (6) | 5.1 (15) |
| **AA/AT** | **34.2 (27)** | **47.6 (78)^†^** | 45.2 (42) | 41.7 (55) | 40.1 (69) | 44.9 (133) |
| **Both sexes** | *n =* 322 | *n =* 308 | *n =* 273 | *n =* 285 | *n =* 595 | *n =* 593 |
| **TT** | 62.4 (201) | 57.8 (178) | 56.0 (153) | **58.9 (168)** | 59.5 (354) | 58.3 (346) |
| **AT** | 31.7 (102) | 35.7 (110) | 41.4 (113) | **34 (97)** | 36.1 (215) | 34.9 (207) |
| **AA** | 5.9 (19) | 6.5 (20) | 2.6 (7) | **7.0 (20)^†^** | 4.4 (26) | 6.7 (40) |
| **AA/AT** | 37.6 (121) | 42.2 (130) | 44.0 (120) | 41.1 (117) | 40.5 (241) | 41.7 (247) |

Statistically significant values are highlighted in bold [Pearson chi square test, 2×3 (TT vs. AT vs. AA) or 2×2 (TT vs. AA/AT) tables].

^*^*p* < 0.1; ^†^*p* < 0.05

**Online Resource 8** Association of small nucleotide polymorphism rs689 alleles and genotypes with type 2 diabetes in the replica case–control study stratified by age at type 2 diabetes diagnosis and sex.

| **Univariate logistic regression models** | | | | | | | |
| --- | --- | --- | --- | --- | --- | --- | --- |
| **Groups** | **Alleles/ Genotypes** | **Dx ≤ 45 years** |  | **Dx ≥ 46 years** |  | **All cases** |  |
|  |  | **OR (95% CI)** | ***p value*** | **OR (95% CI)** | ***p value*** | **OR (95% CI)** | ***p value*** |
| **Alleles (*n =* 2376)** | | | | | | | |
| **Female** |  | *n =* 486/296 |  | *n =* 360/294 |  | *n =* 846/594 | |
|  | T | 1 |  | 1 |  | 1 |  |
|  | A | 1.012 (0.72–1.43) | 0.946 | 1.082 (0.75–1.55) | 0.668 | 1.196 (0.87–1.64) | 0.269 |
| **Male** |  | *n =* 158/330 |  | *n =* 186/260 |  | *n =* 344/592 | |
|  | T | 1 |  | 1 |  | 1 |  |
|  | A | **1.49 (0.94–2.37)** | **0.09** | 0.947 (0.61–1.48) | 0.812 | 1.041 (0.81–1.33) | 0.754 |
| **Both sexes** |  | *n =* 644/626 |  | *n =* 546/554 |  | *n =* 1190/1186 | |
|  | T | 1 |  | 1 |  | 1 |  |
|  | A | 1.185 (0.91–1.54) | 0.203 | 1.022 (0.77–1.35) | 0.88 | 1.104 (0.91–1.33) | 0.310 |
| **Genotypes (*n =* 1188)** | | | | | | | |
| **Females** |  | *n* = 243/144 |  | *n* = 180/153 |  | *n* = 423/297 | |
|  | TT | 1 |  | 1 |  | 1 |  |
|  | AA/AT | 0.896 (0.58–1.37) | 0.614 | 0.891 (0.58–1.38) | 0.605 | 0.909 (0.67–1.23) | 0.539 |
| **Males** |  | *n* = 79/164 |  | *n* = 93/132 |  | *n* = 172/296 | |
|  | TT | 1 |  | 1 |  | 1 |  |
|  | AA/AT | **1.747 (1–3.05)** | **0.049** | 0.867 (0.51–1.48) | 0.602 | 1.218 (0.83–1.78) | 0.311 |
| **Both sexes** |  | *n* = 322/308 |  | *n* = 273/285 |  | *n* = 595/593 | |
|  | TT | 1 |  | 1 |  | 1 |  |
|  | AA/AT | 1.213 (0.88–1.67) | 0.236 | 0.888 (0.63–1.24) | 0.488 | 1.049 (0.83–1.32) | 0.687 |

*CI* confidence interval, *Dx* diagnosis, *n* controls/cases, *OR* odds ratio

Statistically significant values are highlighted in bold. The power (1−β error probability) > 0.99 in all groups with p < 0.05.

# **Online Resource 9** Frequency of rs689 alleles (*n* = 2344) and genotypes (*n* = 1172) in the cross-sectional study stratified by age and sex.

|  | **Controls**  **≤ 47 years** | **Cases Dx**  **≤ 47 years** | **Controls**  **≥ 48 years** | **Cases Dx**  **≥ 48 years** | **All controls** | **All cases** |
| --- | --- | --- | --- | --- | --- | --- |
| **Alleles** |  |  | **Allelic frequency % (*n*)** | |  |  |
| **Female** | *n* = 778 | *n* = 62 | *n* = 800 | *n* = 82 | *n* = 1578 | *n* = 144 |
| **T** | 79.7 (620) | 79.0 (49) | 81.9 (655) | 82.9 (68) | 80.8 (1275) | 81.3 (117) |
| **A** | 20.3 (158) | 21.0 (13) | 18.1 (145) | 17.1 (14) | 19.2 (303) | 18.8 (27) |
| **Male** | *n* = 290 | *n* = 34 | *n* = 268 | *n* = 30 | *n* = 558 | *n* = 64 |
| **T** | 83.8 (243) | 64.7 (22) | 76.1 (204) | 76.7 (23) | 80.1 (447) | 70.3 (45) |
| **A** | 16.2 (47) | **35.3 (12)^‡^** | 23.9 (64) | 23.3 (7) | 19.9 (111) | **29.7 (19)^*^** |
| **Both sexes** | *n* = 1068 | *n* = 96 | *n* = 1068 | *n* = 112 | *n* = 2136 | *n* = 208 |
| **T** | 80.8 (863) | 74.0 (71) | 80.4 (859) | 81.3 (91) | 80.6 (1722) | 77.9 (162) |
| **A** | 19.2 (205) | 26.0 (25) | 19.6 (209) | 18.8 (21) | 19.4 (414) | 22.1 (46) |
| **Genotypes** |  |  | **Genotypic frequency % (*n*)** | | |  |
| **Female** | *n* = 389 | *n* = 31 | *n* = 400 | *n* = 41 | *n* = 789 | *n* = 72 |
| **TT** | 63.2 (246) | 64.5 (20) | 66.3 (265) | 70.7 (29) | 64.8 (511) | 68.1 (49) |
| **AT** | 32.9 (128) | 29.0 (9) | 31.3 (125) | 24.4 (10) | 32.1 (253) | 26.4 (19) |
| **AA** | 3.9 (15) | 6.5 (2) | 2.5 (10) | 4.9 (2) | 3.2 (25) | 5.6 (4) |
| **Male** | *n* = 145 | *n* = 17 | *n* = 134 | *n* = 15 | *n* = 279 | *n* = 32 |

| **TT** | 68.3 (99) | **41.2 (7)** | 58.2 (78) | 66.7 (10) | 63.4 (177) | **53.1 (17)** |
| --- | --- | --- | --- | --- | --- | --- |
| **AT** | 31.0 (45) | **47.1 (8)** | 35.8 (48) | 20.0 (3) | 33.3 (93) | **34.4 (11)** |
| **AA** | 0.7 (1) | **11.8 (2)^‡^** | 6.0 (8) | 13.3 (2) | 3.2 (9) | **12.5 (4)^†^** |
| **Both sexes** | *n* = 534 | *n* = 48 | *n* = 534 | *n* = 56 | *n* = 1068 | *n* = 104 |
| **TT** | 64.6 (345) | 56.3 (27) | 64.2 (343) | 69.6 (39) | 64.4 (688) | **63.5 (66)** |
| **AT** | 32.4 (173) | 35.4 (17) | 32.4 (173) | 23.2 (13) | 32.4 (346) | **28.8 (30)** |
| **AA** | 3.0 (16) | 8.3 (4) | 3.4 (18) | 7.1 (4) | 3.2 (34) | **7.7 (8)^*^** |

*Dx* diagnosis

Statistically significant values are highlighted in bold [Pearson chi square test, 2×3 (TT vs. AT vs. AA) or 2×2 (TT vs. AT/AA) tables].

^*^*p* < 0.1; ^†^*p* < 0.05, ^‡^*p* < 0.01,

**Online Resource 10** Association of small nucleotide polymorphism rs689 alleles (*n* = 2344) and genotypes (*n* = 1172) with type 2 diabetes in the cross-sectional study stratified by age and sex.

| **Groups** | **No. risk alleles** | **Univariate logistic regression models** | | | | | |
| --- | --- | --- | --- | --- | --- | --- | --- |
|  |  | **Dx ≤ 47 years** |  | **Dx ≥ 48 years** | | **All cases** |  |
|  |  | **OR (95% CI)** | ***p value*** | **OR (95% CI)** | ***p value*** | **OR (95% CI)** | ***p value*** |
| **Alleles** |  |  |  |  |  |  |  |
| **Female** |  | *n* = 778/62 |  | *n* = 800/82 |  | *n* = 1578/144 | |
|  | 0 | 1 |  | 1 |  | 1 |  |
|  | 1 | 1.04 (0.55–1.97) | 0.90 | 0.93 (0.51–1.7) | 0.81 | 0.97 (0.63–1.5) | 0.90 |
| **Male** |  | *n* = 290/34 |  | *n* = 268/30 |  | *n* = 558/64 |  |
|  | 0 | 1 |  | 1 |  | 1 |  |
|  | 1 | **2.82 (1.31–6.09)** | **0.008** | 0.97 (0.4–2.37) | 0.95 | **1.7 (0.96–3.02)** | **0.07** |
| **Both sexes** |  | *n* = 1068/96 |  | *n* = 1068/112 |  | *n* = 2136/208 | |
|  | 0 | 1 |  | 1 |  | 1 |  |
|  | 1 | 1.48 (0.92–2.4) | 0.11 | 0.95 (0.58–1.56) | 0.84 | 1.18 (0.84–1.67) | 0.34 |
| **Genotypes** |  |  |  |  |  |  |  |
| **Female** |  | *n* = 389/31 |  | *n* = 400/41 |  | *n* = 789/72 |  |
|  | 0 | 1 |  | 1 |  | 1 |  |
|  | 1 | 0.86 (0.38–1.95) | 0.73 | 0.73 (0.35–1.55) | 0.41 | 0.78 (0.45–1.36) | 0.38 |
|  | 2 | 1.64 (0.35–7.68) | 0.53 | 1.83 (0.38–8.75) | 0.45 | 1.67 (0.56–4.99) | 0.36 |
| **Male** |  | *n* = 145/17 |  | *n* = 134/15 |  | *n* = 279/32 |  |
|  | 0 | 1 |  | 1 |  | 1 |  |
|  | 1 | **2.51 (0.86–7.36)** | **0.092** | 0.49 (0.13–1.86) | 0.29 | 1.23 (0.55–2.74) | 0.61 |
|  | 2 | **28.29 (2.28–351.51)** | **0.0093** | 1.95 (0.36–10.5) | 0.44 | **4.63 (1.29–16.62)** | **0.019** |
| **Both sexes** |  | *n* = 534/48 |  | *n* = 534/56 |  | *n* = 1068/104 | |
|  | 0 | 1 |  | 1 |  | 1 |  |
|  | 1 | 1.26 (0.67–2.37) | 0.48 | 0.66 (0.34–1.27) | 0.21 | 0.9 (0.58–1.42) | 0.66 |
|  | 2 | **3.19 (1–10.23)** | **0.05** | 1.95 (0.63–6.07) | 0.25 | **2.45 (1.09–5.52)** | **0.03** |

*CI* confidence interval, *Dx* diagnosis; *n* controls/cases, *OR* odds ratio Statistically significant values are highlighted in bold.

The power (1−β error probability) > 0.99 in all groups with p < 0.05.

**Online Resource 11.** Association of *TH01* and SNP rs689 alleles with T2D stratified by age at T2D diagnosis and sex (*n =* 3952)^a^.

**Multivariate logistic regression models**

**Loci Alleles**

**Dx ≤ 45 years Dx ≥ 46 years All cases**

**OR (95% CI) *P* value *R*^2^ OR (95% CI) *P* value *R*^2^ OR (95% CI) *P* value *R*^2^**

**Females** *n =* 532 *n =* 504 *n =* 1036

≤ 7R 1

***TH01***

1

**0.043** 0.005

1

**0.0053** 0.010

0.43 **0**

≥ 8R 0.76 (0.58–0.99) 1.46 (1.12–1.9) 1.08 (0.89–1.3)

T 1

**SNP689**

1

0.88 0.005

1

**0.035** 0.015

0.10 0.002

A 1.02 (0.76–1.38) 1.37 (1.02–1.83) 1.19 (0.97–1.47)

**Males** *n =* 468 *n =* 454 *n =* 922

≤ 7R 1

***TH01***

1

**0.012** 0.009

1

**0.0017** 0.014

**< 0.0001** 0.012

≥ 8R 1.45 (1.08–1.93) 1.59 (1.19–2.12) 1.52 (1.24–1.86)

T 1

**SNP689**

1

**0.043** 0.015

1

**0.012** 0.023

**0.0011** 0.019

A 1.38 (1.01–1.87) 1.52 (1.09–2.11) 1.45 (1.16–1.82)

**Both sexes** *n =* 1000 *n =* 958 *n =* 1958

≤ 7R 1

***TH01***

1

0.76 0

1

**< 0.0001** 0.012

**< 0.001** 0.004

≥ 8R 1.03 (0.85–1.25) 1.52 (1.25–1.85) 1.26 (1.1–1.45)

T 1

**SNP689**

1

0.13 0

1

**0.0013** 0.018

**0.0007** 0.008

A 1.18 (0.95–1.47) 1.43 (1.15–1.78) 1.3 (1.12–1.52)

CI, confidence interval; Dx, age at T2D diagnosis; R, repeats; SNP, single nucleotide polymorphism; OR, odds ratio; T2D, type 2 diabetes. *R*^2^, *R*^2^ of Nagelkerke

^a^ A total of 1994 controls (chromosomes) were included: 1070 females and 924 males.

Cases diagnosed with T2D at ≤ 45 years were compared with controls aged ≤ 54 years (*n* = 924) and cases diagnosed with T2D at ≥ 46 years were compared with controls aged ≥ 55 years (*n* = 1070).

The variables were introduced into the model in different blocks, TH01 first block, rs689 second block. The Wald test was used to assess the statistical significance using the Enter method; statistically significant values (p < 0.05) are highlighted in bold.

The power (1−β error probability) > 0.99 for both markers in all groups with p < 0.05.

# **Online Resource 12** Association of *TH01*/rs689 haplotypes with type 2 diabetes stratified by age at type 2 diabetes diagnosis and sex (*n* = 3952)^a^.

| **Univariate logistic regression models** | | | | | | |
| --- | --- | --- | --- | --- | --- | --- |
| ***TH01*/rs689 haplotypes** | **Dx ≤ 45 years** | | **Dx ≥ 46 years** | | **All cases** |  |
|  | **OR (95% CI)** | ***p value*** | **OR (95% CI)** | ***p value*** | **OR (95% CI)** | ***p value*** |
| **Female** | *n* = 532 |  | *n* = 504 |  | *n* = 1036 |  |
| ≤ **7R /T or A** | 1 |  | 1 |  | 1 |  |
| ≥ **8R /T** | **0.53 (0.36–0.77)** | **0.0011** | 1.51 (1–2.29) | 0.052 | 0.9 (0.68–1.19) | 0.45 |
| ≥ **8R /A** | 0.96 (0.7–1.33) | 0.82 | **1.45 (1.07–1.98)** | **0.018** | 1.2 (0.96–1.5) | 0.10 |
| **Male** | *n* = 468 |  | *n* = 454 |  | *n* = 922 |  |
| ≤ **7R /T or A** | 1 |  | 1 |  | 1 |  |
| ≥ **8R /T** | **1.68 (1.05–2.69)** | **0.031** | **1.85 (1.19–2.87)** | **0.0061** | **1.76 (1.28–2.42)** | **0.0006** |
| ≥ **8R /A** | **1.37 (0.98–1.9)** | **0.063** | **1.44 (1.02–2.02)** | **0.037** | **1.41 (1.11–1.79)** | **0.0043** |
| **Both sexes** | *n* = 1000 |  | *n* = 958 |  | *n* = 1958 |  |
| ≤ **7R /T or A** | 1 |  | 1 | 0 | 1 |  |
| ≥ **8R /T** | 0.86 (0.64–1.15) | 0.30 | **1.67 (1.24–2.26)** | **0.0008** | 1.2 (0.98–1.48) | 0.082 |
| ≥ **8R /A** | 1.15 (0.91–1.44) | 0.24 | **1.45 (1.15–1.82)** | **0.0016** | **1.3 (1.1–1.52)** | **0.0016** |

*CI* confidence interval, *Dx* diagnosis, *OR* odds ratio, *R* repeats

^a^ total of 1994 controls (chromosomes) were included (female, *n* = 1070; male, *n* = 924). Cases diagnosed at ≤ 45 years old were compared with controls ≤ 54 years old (*n =* 924) and cases diagnosed at ≥ 46 years old were compared with controls ≥ 55 years old (*n =* 1070).

Statistically significant values are highlighted in bold (Enter method, Wald test). The power (1−β error probability) > 0.99 for both markers in all groups with p < 0.05.

# **Online Resource 13.** Fasting plasma insulin levels in the main case–control study stratified by age at type 2 diabetes diagnosis, sex and *TH01*

and rs689 alleles (*n* = 2564).

| **Fasting insulin (mIU/mL)** | | | | | | | | | | | | | |
| --- | --- | --- | --- | --- | --- | --- | --- | --- | --- | --- | --- | --- | --- |
| **Locus** | **Alleles** | **Controls ≤ 54 years** | | **Cases Dx ≤ 45 years** | | **Controls ≥ 55 years** | | **Cases Dx ≥ 46 years** | | **All controls** | |  | **All cases** |
|  |  | ***n*** | **Median**  **(IQR)** | ***n*** | **Median**  **(IQR)** | ***n*** | **Median**  **(IQR)** | ***n*** | **Median**  **(IQR)** | ***n*** | **Median**  **(IQR)** | ***n*** | **Median**  **(IQR)** |
| **Female** |  |  |  |  |  |  |  |  |  |  |  |  |  |
| ***TH01*** | ≤ 7R | 269 | 7.4 (5–10.5) | 310 | **10.7 (6.5–18)^‡^** | 219 | 6.6 (4.3–9.5) | 346 | **8.9 (5.3–14.4)^‡^** | 488 | 6.9 (4.8–9.8) | 660 | 9.5 (5.7–16.2) |
|  | ≥ 8R | 153 | 6.8 (4.9–11) | 140 | **12.4 (7.2–19.7)^*^** | 71 | 6.3 (5–10.7) | 158 | **10.1 (5.6–16.5)^*^** | 224 | 6.7 (4.9–10.8) | 300 | 10.9 (6.3–19) |
|  | Total | 422 | 7.1 (5–10.6) | 450 | **11 (6.7–18.1)^§^** | 290 | 6.4 (4.3–9.5) | 504 | **9 (5.4–15.3)^§^** | 712 | 6.8 (4.8–10) | 960 | 10.2 (5.8–17) |
| **rs689** | T | 329 | 7.3 (5–10.6) | 343 | **10.8 (6.7–18)^‡^** | 241 | 6.3 (4.3–9.5) | 383 | **9 (5.3–15.2)^‡^** | 570 | 6.8 (4.7–9.9) | 732 | 10 (5.8–16.5) |
|  | A | 93 | 6.5 (4.9–10.3) | 105 | 11.7 (6.6–19.8) | 49 | 7.2 (5.1–11.2) | 119 | 10.2 (5.4–15.5) | 142 | 6.9 (5–10.7) | 224 | 10.7 (5.9–17.9) |
| **Male** |  |  |  |  |  |  |  |  |  |  |  |  |  |
| ***TH01*** | ≤ 7R | 190 | 6.4 (5–10) | 176 | **10 (5.7–18.2)^§^** | 100 | 6.2 (4.4–8.7) | 148 | **6.8 (3.7–10.4)^§^** | 290 | 6.3 (4.7–9.5) | 325 | 8.5 (4.4–14.5) |
|  | ≥ 8R | 72 | 7 (5.2–10.1) | 82 | **10.7 (6.5–19)^‡^** | 40 | 8 (4.2–13.7) | 82 | **8.2 (4.5–12.3)^‡^** | 112 | 7.4 (4.9–11.3) | 165 | 9.1 (5.3–14) |
|  | Total | 262 | 6.9 (5–10) | 258 | **10.1 (6.0–18.2)^§^** | 140 | 6.5 (4.2–10.1) | 230 | **7.1 (3.8–10.7)^§^** | 402 | 6.7 (4.7–10) | 490 | 8.7 (4.8–14) |
| **rs689** | T | 206 | 6.8 (5.1–9.9) | 187 | **10.0 (6.0–17.8)^§^** | 117 | 6.3 (4.5–9.4) | 170 | **6.9 (3.8–10.4)^§^** | 323 | 6.5 (4.7–9.9) | 358 | 8.6 (4.5–13.3) |
|  | A | 56 | 7.0 (4.6–10.3) | 69 | **10.3 (6.3–19)^†^** | 23 | 8.6 (4.2–14) | 60 | **8.2 (3.8–13.2)^†^** | 79 | 7.2 (4.5–12.5) | 130 | 9.4 (5.1–16) |
| **Both sexes** | | | | | | | | | | | | | |
| ***TH01*** | ≤ 7R | 459 | 7.0 (5.0–10.2) | 486 | **10.5 (6.2–18)^§^** | 319 | 6.4 (4.3–9.5) | 494 | **8.1 (4.5–13.9)^§^** | 778 | 6.7 (4.7–9.8) | 985 | 9.2 (5.3–15.9) |
|  | ≥ 8R | 225 | 7.0 (4.9–10.3) | 222 | **12.1 (6.8–19.6)^‡^** | 111 | 6.8 (4.6–11.8) | 240 | **9.1 (5.2–15)^‡^** | 336 | 7 (4.9–10.8) | 465 | 10.3 (5.8–17.1) |
| **rs689** | T | 535 | 7.0 (5.0–10.4) | 530 | **10.8 (6.5–18.0)^‡^** | 358 | 6.3 (4.3–9.5) | 553 | **8.5 (4.7–13.9)^‡^** | 893 | 6.7 (4.7–9.9) | 109  0 | 9.4 (5.4–16) |

| A | 149 | 6.9 (4.8–10.3) | 174 | 11.4 (6.5–19.6) | 72 | 7.4 (4.9–12) | 179 | 9.4 (5.0–15.0) | 221 | 7 (4.8–10.7) | 354 | 10.3 (5.6–17.1) |
| --- | --- | --- | --- | --- | --- | --- | --- | --- | --- | --- | --- | --- |
| **Total** | 684 | 7.0 (5.0–10.3) | 708 | **10.8 (6.5–18.2)^§^** | 430 | 6.4 (4.3–9.9) | 734 | **8.5 (4.8–14.1)^§^** | 1114 | 6.8 (4.8–10) | 145  0 | **9.5 (5.5–16.2)^§^** |

*Dx* diagnosis, *IQR* interquartile range

Cases diagnosed at ≤ 45 years old were compared with controls ≤ 54 years old and cases diagnosed at ≥ 46 years old were compared with controls ≥ 55 years old. Statistically significant values are highlighted in bold (t-test).

^*^*p* < 0.1; ^†^*p* < 0.05, ^‡^*p* < 0.01, ^§^*p* < 0.001
